# A Review of Shared Decision-Making, Published Protocols, and Post-desensitization Strategies in Oral Immunotherapy (OIT)

**DOI:** 10.1007/s11882-024-01132-2

**Published:** 2024-03-05

**Authors:** Susan Laubach, Edwin H. Kim, Matthew Greenhawt, Sally Bailey, Aikaterini Anagnostou

**Affiliations:** 1 Department of Pediatrics, University of California San Diego, San Diego, CA USA; 2 Division of Allergy, Immunology & Rheumatology, Rady Children’s Hospital San Diego, San Diego, CA USA; 3 Division of Pediatric Allergy and Immunology, Department of Pediatrics, University of North Carolina School of Medicine, Chapel Hill, NC USA; 4 Section of Allergy and Immunology, Food Challenge and Research Unit, Children’s Hospital Colorado, Department of Pediatrics, University of Colorado School of Medicine, Aurora, CO USA; 5 Department of Pediatrics, Division of Pediatric Allergy and Immunology, Georgetown University, Washington, DC USA; 6 Allergy Associates of Northern Virginia, Arlington, VA USA; 7 Department of Pediatrics, Section of Immunology, Allergy and Retrovirology, Texas Children’s Hospital, Houston, TX USA; 8 Section of Allergy, Immunology & Retrovirology, Baylor College of Medicine, Houston, TX USA

**Keywords:** Food allergy, Oral immunotherapy, Shared decision-making, Dose escalation, Maintenance therapy

## Abstract

**Purpose of Review:**

The aim of this review is to highlight key published oral immunotherapy (OIT) protocols and post-desensitization strategies for the major food allergens and to cover important concepts to consider when evaluating OIT for food-allergic patients. Shared decision-making should help identify patient and family values which will help influence the type of evidence-based protocol and maintenance strategy to use.

**Recent Findings:**

With food OIT emerging as a treatment option, there is a pressing need for patients, physicians, and other providers to have a nuanced understanding of the management choices available to them. There are now randomized controlled trials (RCT) of OIT for peanut, egg, milk, and wheat, and reports of cohorts of patients who have undergone OIT for tree nuts and sesame clinically. The current published protocols contain significant diversity in terms of starting dose, build-up schedule, maintenance dose, and even the product used for desensitization. Emerging data can help direct the long-term maintenance strategy for patients on OIT.

**Summary:**

Based on patient and family values elicited through the shared decision-making process, an OIT protocol may be selected that balances the level of desensitization, potential side effects, frequency of clinic visits, and potential to induce sustained unresponsiveness, among other factors. Once maintenance dosing is reached, most patients will need to maintain regular exposure to the food allergen to remain desensitized. The option to transition to commercial food products with equivalent amounts of food protein as the OIT maintenance dose would simplify the dosing process and perhaps improve palatability as well. Less frequent or decreased OIT dosing can provide practical benefits but may affect the level of desensitization and safety for some patients.

## Introduction

The first report of oral immunotherapy (OIT) for food allergy was published over 100 years ago [1], although the bulk of OIT research derives from the past 20 years. The FDA approved the first OIT product to treat peanut allergy in 2020, although the Joint Task Force of the American Allergy & Immunology organizations does not yet have a practice parameter or guidelines on OIT. The European Academy of Allergy and Clinical Immunology recommends that OIT be offered to patients with milk, egg, or peanut allergy because they conclude that “trials have found substantial benefit…in terms of efficacy” for OIT to these foods [2]. The Canadian Society of Allergy and Clinical Immunology recommends that OIT be available to patients who want it, through shared decision-making with their Allergist/Immunologist [3], while the Australasian Society of Clinical Immunology and Allergy does not routinely recommend OIT.

There is strong evidence in favor of the safety and efficacy of OIT. In a recent meta-analysis, 8 high-quality randomized clinical trials (RCTs) for peanut, 6 for hen’s egg (HE), and 4 for cow’s milk (CM) were identified, and the data supports the efficacy of OIT to induce desensitization for peanut, HE, and CM, and sustained unresponsiveness (SU) for peanut and HE [4]. Adverse reactions during OIT occur more frequently than during avoidance; however, these reactions are typically mild, and few patients discontinue OIT because of them [5]. Small studies on multiple-food OIT have suggested a similar safety and efficacy profile [6, 7]. The effects of OIT may be dependent on the starting or maintenance dose (or goal), duration, and frequency of dosing as well as patient-specific factors, such as age and food-specific IgE levels [8].

The goal of this review is to discuss (a) important concepts to consider during shared decision-making with patients/families, (b) representative-published OIT protocols for specific foods, and (c) the course of OIT after maintenance is reached. Other forms of food immunotherapy and emerging food allergy treatments are outside the scope of this review but have been reviewed elsewhere [9], as have practical considerations for the implementation of OIT in clinical practice [10].

## Addressing Misinformation in Food OIT

Because food allergy treatment is such a new paradigm shift in management, there is a dearth of evidence-based materials to help educate patients and very limited evidence regarding baseline patient knowledge and understanding of OIT [11, 12]. Food-allergic caregivers have high levels of trust in on-line information, but limited ability to discern if sources are reputable or the information is valid [3, 11, 13, 14]. Multiple online sources exist that describe or discuss food OIT and related issues, without any medical oversight or use of evidence-based information. There is a risk that low-quality and inaccurate information may help cement confirmation bias and other cognitive biases, which can negatively impact patients, and potentially motivate them to discredit reliable information if it contradicts their previously held beliefs [15–17]. For instance, information or advice on OIT from a celebrity figure may carry more influence than from a medical professional. Or an individual may only accept as true, information that supports their existing belief that OIT is curative. Recent studies have highlighted the poor quality of information found online for food allergy and anaphylaxis [18]. Altogether, these issues and potential negative influences on perception are all compounded by a lack of reputable and evidence-based educational information for the caregivers. No standardized OIT educational materials exist, and only two decision aids related to food OIT are currently available [19, 20].

Clinicians are tasked with staying up-to-date with current evidence-based information and communicating such evidence in a clear manner to patients. However, this traditional information exchange is now rivalled by online sources of information which are often unvetted and can be biased, outdated, incorrect, or lacking in context [17, 21]. For example, food OIT is often presented as a curative form of treatment or discussed in online forums as part of a personal experience or patient journey that may be completely irrelevant to a different patient. Additionally, incorrect advice may be provided on how to address OIT-related medical issues by individuals who may have no medical background or expertise in this area. Individuals often place high trust in non-medical professionals; anecdotal stories may garner attention and emotional responses though these experiences do not generalize or apply to the majority of cases [16, 18]. Clinicians are at risk of having their evidence-based OIT expertise muted by these unvetted online sources of information. To best help our patients, clinicians need better educational materials on food OIT which can be easily implemented into daily clinical practice.

## Shared Decision-Making in Food Allergy Therapy

With food OIT emerging as a therapy option, there is a pressing need for patients to have a nuanced understanding of their diagnosis and the choices available to them. Shared decision-making is a key component of any patient-physician interaction. It is a mutual process during which patients and allergists explore food OIT-related goals and risks/benefits of different available treatment options (for example, avoidance versus OIT), with the aim to provide the option that best fits each individual’s needs [22, 23]. In addition to the traditional option of strict food allergen avoidance and now the option of food OIT, other options are being developed, including biologics (e.g., anti-IgE therapy) and other forms of immunotherapy [15]. As other therapies become available, comparative-efficacy research will be useful, and a good understanding of different options will be required from patients and healthcare professionals, including information on risks and side effects for each treatment, eligibility criteria, time commitment, and cost. All these areas can be addressed during the OIT consultation, with the physician carefully describing the process of OIT, the risks and benefits of this intervention, in addition to how OIT performs compared with food avoidance in terms of protection from accidental exposures, severity of allergic reactions, and quality of life outcomes. The family’s and patient’s aims and preferences need to be explored in detail so the physician can obtain a better understanding of the goals of therapy and how OIT would fit in the family’s everyday life. The physician will also need to ensure the patient is eligible for this therapy; uncontrolled asthma and a history of EoE, for example, would be contraindications to this treatment.

Decision aids are tools that can help caregivers in the shared decision-making process to clarify their values and understand their therapy options, including expected OIT outcomes, side effects, and how treatment impacts overall prognosis [19, 24]. They are not stand-alone tools for making a decision but are meant to supplement the allergist’s encounter. Only two decision aids exist currently in food allergy; both address food OIT [19, 20]. Clinicians can use these tools during or after the consultation, and patients can spend time at home familiarizing themselves with the decision aids and bringing questions and discussion points back to the allergist at their next visit. Creation of additional such tools will help standardize the information provided, which can reduce clinician-level variability in what families are told, reducing the potential for bias and ensuring every patient receives high-quality, consistent education about their disease state and management options.

## Protocols from Key Research Studies of OIT

Once a patient or caregiver decides to pursue OIT, a specific protocol should be selected. Many high-quality studies exist for peanut, egg, and milk OIT, and while some foods have only been studied in a few trials or observational studies, this review includes many of the relevant, existing studies for the top allergens. There is significant diversity in published protocols, in terms of starting dose, build-up schedule, maintenance dose, and even the food product used for desensitization. Based on the patient and family values elicited through the shared decision-making process, an OIT protocol may be selected that balances the level of desensitization, potential side effects, frequency of clinic visits, and potential to induce SU, among other factors. A summary of representative OIT protocols for the most common food allergens is shown in Table 1. The included studies are not an exhaustive list of all published studies, but seminal RCT and open-label studies that highlight the diversity of protocols. When studies used similar protocols, we highlight the study with the strongest data; for example, we include a Phase 3 double-blind placebo-controlled (DBPC) RCT [25] rather than Phase 1 or 2 trials. We tried to include reports that highlight the diversity of starting dose and maintenance doses. When less than 3 RCT exist for a food, we also include non-randomized reports. The reader is directed to review the specific studies or one of several meta-analyses of HE, CM, and peanut OIT for more granular details about these trials [4, 5, 26].

**Table 1:** Summary of representative OIT protocols for the most common food allergens

**Study/type**	**Number of active OIT**	**Age in years median (range)**	**sIgE (kU/L) median (range)**	**Product used**	**Starting dose (mg protein)**	**Initial dose escalation (IDE)**	**Steps to maintenance (after IDE)**	**Frequency of build-up**	**Maintenance (mg protein)**	**Time to maintenance (weeks)**	**Percent reaching maintenance**	**Anaphylaxis or moderate/severe reaction rate due to OIT**	**EoE symptoms reported proportion of subjects**
**(a) Peanut**													
**Anagnostou 2014;** RCT cross-over	99 total: 49 active, 50 control cross-over to active	12.3 (8.1–16.3)	37.9 (0.35–3649)	12% lightly roasted, partially defatted peanut flour (Golden Peanut Co, Alpharetta, GA)	2 mg	No	9	Every 2–3 weeks	800	24	84–91%	1% (1/99) subjects Epi [0.01% (2/17,793) doses]; 22% (21/99) wheezed [0.41% (7/17,793) doses]	NR
**Vickery 2017;** DBPC RCT preschool	37 active: 20 low-dose, 17 high-dose, 154 matched controls	2.3 (1.8–2.9)	14.4 (3.4–48.6)	12% lightly roasted, partially defatted peanut flour (Golden Peanut Co, Alpharetta, GA)	0.1 mg	7 doubling doses every 30 min up to 6 mg	17 low, 27 high	Every 2 weeks	300 vs 3000	NR	81% (30/37) total; 85% (17/20) low; 76% (13/17) high	17% (36/211) moderate, no severe reactions. 3% of subjects (0.5% of events) required Epi	5.4% (2/37) subjects withdrew due to chronic GI symptoms, 1 with EoE on biopsy
**PALISADE 2018;** DBPC RCT	372	(4–17)	61 (17–179 IQR)	AR101 characterized peanut flour with 50% protein content (mg) (drug)	0.5 mg	5 doubling doses up to 6 mg	11	Every 2 weeks	300	24	79% (294/372)	4.3% vs 0.8% severe; 14% vs 6.5% Epi	4.3% (16/372) withdrew due to chronic GI symptoms; 1 with EoE on biopsy
**Blumchen 2019;** DBPC RCT	31	6.6 (4.8–9.8 IQR)	89.5 (6.9–217 IQR)	Light roasted peanut flour (12% fat, 50% protein) from the Byrd Mill Company (Ashland, Va) mixed into a “ready-to-eat” chocolate pudding	0.5–30 mg based on reactive dose (RD) during OFC	OFC starting with 3 mg, 1 dose every 2 hours up to 300 mg on day 1, then up to 3000–4500 mg on days 2 and 3 if tolerated	33	Every 2 weeks	125–250 (based on reactive dose during entry OFC < or ≥ 300 mg)	52 (40–56)	50% reached maintenance; 74% (28/31) tolerated 300 mg OFC	23% (7/30) subjects vs. 16% (5/31) controls with Grade 4–5; 0% Epi	NR
**Davis 2022;** Open-label high-dose	15	8.7 (6.2–12.2)	62.5(0–568)	12% lightly roasted, partially defatted peanut flour (Golden Peanut Co, Alpharetta, GA)	1.8–750 mg based on RD during OFC	DBPC OFC with 10 doses starting at 0.5 mg up to 2000 mg cumulative to determine RD, then starting dose determined by RD, minimum 1.8 mg	26	Every 2 weeks	3900	52 (50–71)	71% (12/17)	53% (8/15) subjects Epi; 2.5% (30/1188) doses severe	0%
**Jones 2022 (IMPACT trial)**; DBPC RCT preschool	96	3.3 (2.6–3.7 IQR)	54 (28–192)	12% lightly roasted, partially defatted peanut flour (Golden Peanut Co, Alpharetta, GA)	0.1 mg	5 doses every 30 min up to 6 mg	NR	Every 2 weeks	2000	30	61% (59/96 ITT)	22% (v 0%) required Epi, 42% (v 8%) moderate, 5% (v 0%)severe	3% (3/96)
**(b) Hen’s egg (HE)**													
**Burks 2012**; DBPC RCT	40	(5–18)	10.3 (3.7–231.1)	Raw egg white (EW) powder (Deb-El Food Products): 0.8 mg protein in 1 mg powder	0.08 mg	Dosing began with 0.1 mg raw EW powder, followed by an approximate doubling every 30 min up to 50 mg. Median IDE dose 18.5 (range 6–50) mg egg powder	NR	q2 weeks	1600	40	45% reached 2000 mg by 10 months, 82% by 22 months (15% discontinued OIT due to adverse events)	2.5% (1/40)	7.5% (3/40) withdrew before 5.5 months due to intermittent abdominal pain and/or vomiting
**Dello Iacono 2013**; RCT	10	6.6 (5–10.2)	23.30 (8.9–35.5)	Undiluted raw HE emulsion (45 mL of raw HE and 150 mL of amino acid-based infant formula) flavored with vanilla	1 drop (0.015 mL) undiluted raw HE emulsion	No	26 slowly escalating doses of raw HE at home (1 drop–1 mL = 10–50%) alternating with 5 dosage doubling in hospital	Weekly at home or in the hospital	40 mL of raw HE emulsion roughly corresponds to a small egg	24	0% active reached maintenance. Median MTD was 20 mL; (range: 5–30)	0% grade 5 reactions; 75% (40/53) had grade 3–4 reactions; 30% controls had grade 3–4 reactions to accidental ingestion per Sampson’s classification	NR
**Meglio 2013**; RCT	10	8.4 (4–17)	11.3 (5.6–150) ovalbumin sIgE	Raw HE (mixed EW and yolk)	0.27 mg	No	181	1–6 days at home;* initial dose administered under observation*	13,600 per week; (corresponds to 3 eggs per week)	30.7 (25–41) mean (range)	80% vs 20%	0%	NR
**Escudero 2015**; RCT	30	7.8 (2.9) mean (SD)	6.4 (1–245)	3600 mg of dehydrated EW (OVOSEC SA, Valladolid, Spain) is equivalent to one medium size EW and contains at least 78% protein (2808 mg)	0.08 mg	12 doses given every 20 min up to 140 mg (cumulative dose of 280 mg) EW protein	9 starting with MTD	Weekly	Maintenance: 1 egg every 48 h plus no egg restriction in the diet	32.5 days (median) since most children tolerated a cumulative dose of 280 mg during IDE. Per protocol 9 weeks. 3 children needed > 12 weeks	93% (28/30) reached maintenance	3% (1/30) required epinephrine for rhinitis, urticaria, and mild resp distress during build-up after a 2404 mg dose. 6.3% (5/79) of reactions in build-up phase were classified as “respiratory distress”	Two children (7%) were withdrawn from the study for non-severe repeated allergic reactions (abdominal pain and vomiting) during build-up. These two patients had EW-sIgE levels of 5.6 and 245 kU/L, respectively
**Akashi 2017;** RCT	18	5 (4–11)	19.5 (7.2–46.2)	Dry HE powder (425 mg egg protein in 1000 mg dry HE powder) based on protocol by Patriarca (2003), per authors	0.0425 mg	No	26	q3–4 days at home; *no observed doses*	1700; (equivalent to approx. 1/4 raw egg)	NR	56%	5% (1/18); one 4y subject had 2 episodes of anaphylaxis to OIT and withdrew	NR
**Pérez-Rangel 2017;** RCT high-dose rush OIT	19	10.4 mean (2.6 SD)	5.6 (0.28–1735) EW sIgE	Dehydrated EW (OVODES NM, Nutrición Médica SL, Madrid, Spain)	175.5 mg EW protein (range, 0.3–1404 mg) median initial dose of the build-up phase. Minimum dose 0.3 mg EW protein	Rush OIT: minimum 0.03 mg EW protein for those reacting to 1st dose of DBPCFC (start dose based on MTD in DBPCFC), 2–5 doses per day over 5 days to a dose of 2808 mg EW protein	11.6 (9.39) mean (SD) of doses per patient (range, 2–42 doses) for rush protocol	1 hour	2808	3 days (1-14)	94% (31/33) reached maintenance, 91% continued 5 months	6% (2/33) of subjects 1.3% of doses	3% (1/33) discontinued due to chronic GI sx
**Itoh-Nagato 2018;** RCT rush OIT multicenter, parallel-group, delayed start	45; 23 early-start; 22 late start	7 (5–12)	35.9 (1.2–201.6)	1000 mg raw EW powder = 8000 mg EW	1/10th of RD during DBPCFC: 10 mg (1–50) EW powder	Starting with 1/10 of RD, doses increased 120–150% every 30 min for 3–5 doses per day, up to 1000 mg of EW powder	None. Rush protocol only—protocol not published	30 min	1000 mg EW powder plus 1 scrambled egg every 1–3 days, plus ad lib	NR	100% tolerated > 1000 mg EW powder after rush OIT and 3 months of maintenance	4.4% (2/45) subjects discontinued due to anaphylaxis (1 during DBPC OFC)	8.8% (4/45) discontinued due to chronic GI sx
**Martín-Muñoz 2019 SEICAP 1;** RCT; OIT (pattern based on center) vs control	88; 12/25 control started OIT after 12 mo observation	7 (6–9)	6.44 (0.22–2045.00)	Pasteurized EW from Guillen, Valencia, Spain (Jurado-Palomo, 2010)	0.11 mg	Starting with 1 mL of a 1/1000 water solution of pasteurized EW, double doses every 30 min over 8 doses up to 0.4 mL of undiluted pasteurized EW (44 mg protein)	96 (7–329) median (range) total doses during buildup phase	Daily and/or weekly	3300; 30 mL pasteurized EW every 1–2 days	14 (1–47) days median induction period	84.2% (64/76) vs 16% (4/25) controls	3.5% (15/420 reactions) severe	NR
*Pattern 1: build-up weekly in-hospital PLUS daily at home*	*21*	6.9 (6.1–8.7)	2.90 (0.40–25.10)	-	0.11 mg	*See above*	65 (27–154); 9 (4–22) hospital; 55 (23–136) home	30% weekly observed plus 5% daily home build-up	3300; 30 mL pasteurized EW every 1–2 days	9.3 (3.9–22)	96% (25/26)	Epi: 3.8% (1/26)	NR
*Pattern 2: build-up weekly in hospital only*	*55*	7 (6–9)	7.4 (0.2–227.0); *p* = 0.007	-	0.11 mg	*See above*	125 (7–329); *p* = 0.02	30% weekly observed build-up	3300; 30 mL pasteurized EW every 1–2 days	18 (1–47) *p* < .000	75% (47/62); *p* = 0.01	Epi 9.7% (6/62); *p* < 0.05	NR
**(c) Cow’s milk (CM)**													
**Longo 2008;** RCT	30	5–17	> 85 kUA/mL	CM	5 drops of 1:10 CM dilution	36 doses over 10 days in hospital of 7 dilutions of CM starting with 5 drops of 1:10 dilution, up to 20 mL undiluted CM	> 100	Every other day at home if tolerated	150 mL	52	36%	7%	NR
**Skripak 2008;** DBPC RCT with subsequent open label for placebo	19; 13 DBPC RCT [6 open-label, minimal data reported]	9.3 (SD 3.3, range 6–17)	34.8 kUa/L (range, 4.86–314 kUa/L)	Dry nonfat powdered CM	0.4 mg	8 doses over 1 day starting with 0.4 mg of CM protein, approximately doubling doses every 30 min to a maximum of 50 mg (cumulative dose, 98.7 mg). Participants had to tolerate a minimum dose of 12 mg	8	Every 7–14 days	15 mL (33 mg/mL)	8 to 16	92% (12/13) of active and 100% open-label	31% (*n* = 4) of subjects (1% of doses)—2 during IDE and 2 during home-dosing	NR; 2 subjects in active group accounted for 2/3 of all GI-related symptoms, both mild abdominal pain not requiring treatment
**Pajno 2010;** single-blind placebo-controlled RCT	15	4–12	32.7 (8.8–124.6)	CM	1 drop of 1:25 CM	No	18	Weekly	200 mL	18	77%	20% (3/15)	NR
**Martorell 2011**; RCT	30	6.6 (6.5–8.0)	15 (3.35–48.7)	CM	1 mL of 1:100 CM	10 doses over 2 days of 3 dilutions CM starting with 1 mL of 1:100 dilution, up to 2.5 mL undiluted CM	16	Weekly	200 mL	16	90%	7%	NR
**Salmivesi 2012;** DBPC RCT with subsequent open label for placebo	28; 18 DBPC RCT; [10 open label]	6–14	0.7 to > 400 IU/mL	Pasteurized 2.5% fresh CM	0.06 mg; 1 drop of 1:25 CM (20 drops = 1 mL)	No	136	Every 1–3 days at home, weekly in office	200 mL	23	86%	0%	7% (2/28) discontinued OIT on days 11 and 42 due to abdominal pain
**Lee 2013;** RCT	16	7–12 mo	16.8 +/− 12.1 (mean)	CM	16.7 mg; 0.5 mL CM	5 doses in 1 day q 30 min starting with 0.5 mL up to 2 mL	22	Weekly	200 mL	24	88%	13%	NR
**Nagakura 2021;** RCT	33; 17 heated milk HM; 16 unheated milk UM	HM: 7.6 (5.2–11.2); UM: 6.1 (5.3–10.8)	HM: 56.0 (4.3–2630); UM: 55.2 (12.5–745)	HM: CM powder, prepared by heating CM at 125 °C for 30 sec and spray-drying for 3 sec. 1 packet of CM powder dissolved in 30 mL = 6 mL CM (0.2 mL CM / 1 mL HM); UM undiluted CM	Start at half the RD of the DBPCFC. 0.1 mL CM or 0.5 mL HM	No	8	Monthly at home	3 mL CM / 15 mL HM	32 to 52	HM 94%; UM 75%; (*p* = .17) achieved desensitization to the equivalent of 3 mL CM	Anaphylaxis: 0.02% doses HM; 0.05% doses UM; Moderate/severe sx: 0.7% doses HM, 1.4% doses UM; respiratory symptoms: 1.2% doses HM, 2.6% doses UM; *p* < .0001	3% (1/33, from UM group) discontinued OIT because of eosinophilic esophagitis at 1 month
**(d) Wheat**													
**Sato 2015;** open label rush high-dose OIT	18	9 (5.9–13.6)	> 100 (2.92–> 100)	200 g boiled udon (Japanese wheat noodles that contain 5200 mg of wheat protein; Tablemark, Co, Ltd, Tokyo, Japan)	50 mg	10 steps starting with an OFC at 50 mg building up to 5200 mg wheat protein. Dose increased q5h BID over 5 days	20	Weekly at home	5200	20 (up to 2 years)	89% (16 /18)	5.5% (1/18) subjects treated with Epi; 0.04% (3/5776) doses considered severe	5.5% (1 of 18) withdrew due to chronic abdominal pain
**Nowak-Węgrzyn 2019;** RCT low vs high dose	46; 23 low-dose; 23 placebo cross-over to high dose	8.7 (4.2–22.3)	88.4 (5.2 to 101)	Vital wheat gluten (VWG) is a high-protein content (71%) substance since wheat flour is too bulky. Administered in vials, capsules, scoops—depending on the dose	0.07 mg	8 doses	15 steps to low-dose. 18 steps to high-dose. Including IDE: 23 steps to low-dose, 26 steps to high-dose	2 weeks	1445 (low-dose); 2748 (high-dose) wheat protein	Up to 44; 34 for low-dose; 40 for high-dose; median	83% for low-dose, 57% for high-dose after 1 year	Low-dose year 1: **0.04% of doses** caused severe AR, and **0.08% of doses** required epinephrine; year 2 none were severe or required epi. High-dose: **0.01% of doses** were severe, and **0.07%** were treated with epinephrine	4.3% (1/23) low-dose withdrew due to mild abdominal pain and vomiting. 9.5% (2/21). High-dose withdrew due to EoE or intermittent GI sx despite PPI and H2 blocker
**Nagakura 2020;** Prospective observational of low-dose OIT with historical control	16	6.7 (5.8–10.7)	293 (4.5–3340)	2 g boiled udon noodles (5 cm) contain 53 mg wheat protein	2.7 mg	Starting with half the threshold dose of OFC, 8-step increase to 53 mg. The first 5 steps occurred once daily in-hospital, subsequent increases at home every 5 days	8 (including IDE)	Daily × 5 days in hospital (IDE), then every 5 days at home	53 mg	NR	88% (14/16)	31% (5/16) subjects; 0.1% (7/5857) vs 0% doses; 5 of 7 occurred during build-up	0%
**Nguyen 2023;** Retrospective review of multi-food OIT in academic center	4	NR	NR	Wheat flour	1.3 mg	Each food was introduced separately during an IDE. First 3 doses administered every 20 min up to 10 mg wheat protein	10 to 16	2 weeks	2000	NR	0% (0/4) reached maintenance by publication	0%	NR
**(e) Tree nuts and seeds**													
**Walnut**													
**Elizur 2019;** Prospective cohort with observational control	55	7.9 (6–9.7)	6.4 (2.5–19.8)	Walnut flour (Wellbees, Monsey, NY) finely ground in a coffee grinder until a homogenous paste was generated, mixed with mineral water 25 mg/mL. Whole walnuts used after 150 mg protein dose reached	0.1 mg	2 consecutive days with 7–8 doses given every 10–30 min up to 300 mg	NR	Monthly	4000, then maintained on 1200	24	89% (49/55)	20% Epi in clinic (< 1% doses), 15% Epi at home (< 1% doses)	5% (3/55). Dose decreased, then built up to maintenance
**Erdle 2023;** Prospective, registry of multi-tree nut OIT in preschool children	31; 19 single TN; 12 multi-TN	1–5	≥ 0.35 kU/L or ≥ 5 if never ingested	5 g protein /30 g flour; 3g protein/240 mL milk	1–10 mg	No	8 to 11	2–4 weeks	300	NR	97% (30/31)	3% (1/31)	3% (1/31, on walnut/cashew OIT)
**Nguyen 2023**; Retrospective review of multi-food OIT in academic center	27	NR	33.5 ± 28	Flour	1.3 mg	Each food was introduced separately during an IDE and build-up. First 3 doses administered every 20 min up to 10 mg of walnut	10 to 16	2 weeks	~300	22	89% (24/27)	0%	1 patient with recurrent vomiting had a negative evaluation for EoE (unclear which foods)
**Cashew**													
**Elizur 2019;** Prospective cohort with observational control	50	8 (6.2–10.8)	4 (1.5–8)	Cashew flour (Wellbees, Monsey, NY - 800 mg flour contains 144 mg protein) or whole cashews	0.1 mg	3 consecutive days with 5–8 doses given every 10–30 min up to 3960 mg or until MTD identified	18	Repeat IDE monthly starting with MTD	4000; then maintained on 1200	52 (12–228)	88% (44/50)	18% clinic, 6% home	4% (2/50). Dose decreased, then built up to maintenance
**Erdle 2023;** Prospective, registry of multi-tree nut OIT in preschool children	66; 58 single TN; 8 multi-TN	(1–5)	≥ 0.35 kU/L or ≥ 5 if never ingested	5 g protein /28 g flour 4 g protein/240 mL milk	1–10 mg	No	8 to 11	2–4 weeks	300	NR	94% (62/66)	7.5% (5/66)	3% (2/66, 1 on walnut/cashew, 1 on cashew alone)
**Nguyen 2023**; Retrospective review of multi-food OIT in acadmic center	35	NR	27.7 ± 31.4	Flour	0.8 mg	First 3 doses administered every 20 min up to 3.2 mg cashew	10 to 16	2 weeks	~300	27	83% (29/35)	22% (8/24) required Epi during IDE. 1 severe reaction to IDE requiring 2 epi and hospitalization	NR
**Hazelnut**													
**Moraly 2020;** Retrospective	100 included in analysis; 168 started	5 (3–9)	5.5 (IQR 1.7–20.9)	Ground hazelnut	0.7 mg	Increasing doses (twice the cumulative dose) were administered every 20 min to a maximum cumulative dose of 1635 mg or RD	11 steps to maintenance	Increase monthly from 1/10th to 1/8th to 1/6th to 1/4th to 1/3rd to 1/2 RD up to 1635 mg	416	24	34% at 6 months	0%	0%
**Erdle 2023**; Prospective, registry of multi-tree nut OIT in preschool children	8; 1 single TN; 7 multi-TN	(1–5)	≥ 0.35 kU/L or ≥ 5 if never ingested	5 g protein /28 g flour 2 g protein/240 mL milk	1–10 mg	No	8 to 11	2–4 weeks	300	NR	100%	0%	0%
**Nguyen 2023**; Retrospective review of multi-food OIT in academic center	10	NR	30 ± 27.5	Flour	2 mg	First 3 doses administered every 20 min (final dose NR)	10 to 16	2 weeks	~190	26	100% (10/10)	17% (2/12) required Epi during IDE	NR
**Almond**													
**Erdle 2023;** Prospective, registry of multi-tree nut OIT in preschool children	2 (both multi-TN)	1–5	≥ 0.35 kU/L or ≥ 5 if never ingested	6 g protein /28 g flour 5 g protein/240 mL milk	1–10 mg	No	8 to 11	2–4 weeks	300	NR	100%	0%	0%
**Nguyen 2023;** Retrospective review of multi-food OIT in academic center	2	NR	19.7 ± 14.1	Flour	2 mg	First 3 doses administered every 20 min up to 10 mg almond	10 to 16	2 weeks	~230	17	100% (2/2)	0%	NR
**Macadamia**													
**Erdle 2023;** Prospective, registry of multi-tree nut OIT in preschool children	1 (single TN)	(1–5)	≥ 0.35 kU/L or ≥ 5 if never ingested	2 g protein /28 g flour 1 g protein/250 mL milk	1–10 mg	No	8 to 11	2–4 weeks	300	NR	100%	0%	0%
**Sesame**													
**Nachshon 2019;** Open label OIT	60	7.5 (5.8–11.6)	NR	< 240 mg doses used standardized high-protein sesame extract (HPSE), then Tahini (1 g = 240 mg sesame protein)	0.3 mg	2 consecutive days with up to 7 doses given every 15–60 min up to 4800 mg sesame protein or until the RD identified. Then 2 additional days with 2–3 doses per day confirming MTD for home dosing.	14 doses total, including IDE	Repeat IDE monthly × 3 starting with MTD, then monthly increase by 50%	4000 then maintained on 1200	26 (15–52)	88% (53/60), 95% partial (240 to < 4000 mg)	16.7% pts in hospital, 8.3% pts at home needed Epi	5.7% (3/60). Dose decreased then built up to maintenance
**Nguyen 2023;** Retrospective review of multi-food OIT in academic center	12	NR	24.4 ± 16.9	Flour	2 mg	First 3 doses administered every 20 min up to 7 mg sesame	10 to 16	2 weeks	375	28	75% (9/12)	25% (4/16) required Epi during IDE. 1 with severe anaphylaxis (abdominal pain, facial flushing, and change in demeanor) requiring Epi × 3 and transfer to PICU	NR

Abbreviations: *AR* adverse reaction, *DBPC* double-blind placebo-controlled, *EoE* eosinophilic esophagitis, *GI* gastrointestinal, *IDE* initial dose escalation, *IQR* interquartile range, *ITT *Intention to treat, *MTD *maximum tolerated dose, *NR *not reported, *OFC* oral food challenge, *RCT* randomized controlled trial, *SD* standard deviation, *sIgE* specific IgE, *SPT* skin prick test, *yrs* years

### Peanut OIT Protocols

Of all food OIT, the most high-quality data exists for OIT to peanut [4]. The variety in protocols is more narrow, so we highlight 5 RCT and one open-label high-dose study for review [25, 27–31]. Protocols uniformly used dry-roasted, partially defatted peanut flour from Golden Peanut (Alpharetta, Ga) [27–29, 31], Byrd Mill (Ashland, Va) [30, 32], or the now FDA-approved characterized peanut powder known as AR101 [25, 33], mixed in a semi-solid vehicle such as chocolate pudding or applesauce. Most studies excluded subjects with a history of severe anaphylaxis with hypoxia or hypotension, although some studies did not [30, 31]. Eosinophilic esophagitis (EoE) was an exclusion criteria in some studies [25, 27, 28], and the discontinuation rate due to chronic GI symptoms was < 6% where reported [25, 27, 29, 34]. Subjects were typically school-aged, although two studies focused on preschool children which both suggested that SU may be more likely in this age group [29, 34]. Two studies included some adults but reported minimal data specific to this population making it hard to analyze for statistical significance [25, 35].

Most protocols included an initial dose escalation (IDE) starting with 0.1 mg or 0.5 mg peanut protein and doubling doses every 30 min up to 6 mg [25, 28, 29]. Others conducted an oral food challenge (OFC) to determine the reactive dose (RD), on which the starting dose for the build-up phase was based [27, 30], and one protocol started with a single dose of 2 mg without an IDE or OFC to start desensitization [31]. Build-up uniformly occurred under observation every 2 weeks, with daily dosing at home after the dose was tolerated in clinic, without premedication, where reported [25, 27, 29, 31]. The maintenance dose clustered into either low-dose (≤ 300 mg) [25, 29, 30] or high-dose (2000–4000 mg) [27–29], although one protocol used an intermediate maintenance dose of 800 mg [31]. One study in preschool children compared 300 mg vs 3000 mg maintenance doses and found similar end-point efficacy with a lower drop-out rate for those on the lower maintenance dose [29]. Build-up lasted 6–12 months, but the duration did not necessarily correlate with the final maintenance dose as evidenced by one study of low-dose OIT in which only 50% of subjects reached the maintenance dose of 125–250 mg peanut protein by 14 months [30].

The majority of subjects experienced adverse events (AEs) during build-up, although most AEs were mild and were often similar to the rate reported by controls. The criteria used to grade AEs varied; however, severe AEs were reported in 0–23% of subjects, and 0–53% of subjects were treated with epinephrine during OIT. In exit OFCs from all studies, most subjects could tolerate higher doses than their maintenance dose. One study was designed to investigate the maximum tolerated dose (MTD) after OIT and found that the majority of subjects were able to tolerate 3–4 times their maintenance dose of 3900 mg [27].

### Egg OIT Protocols

Over a dozen studies have been published on HE OIT in children, and we chose 8 RCTs to represent a spectrum of published protocols [36–43] which all included DBPCFCs before and after desensitization to test efficacy. Some excluded subjects with a history of severe reactions [37, 39, 41, 42]. One study only enrolled subjects with severe allergy [36], and none of these subjects were able to reach maintenance [36]. Protocols used either dehydrated egg powder [37–39, 41, 42] or raw egg [36, 40, 43]. Most used egg white (EW) [37, 38, 40–42], while others used a mix of EW and yolk [36, 39, 43]. Please see Table 1 for details of the product used for OIT in each trial.

The starting dose ranged from 0.03 to 0.27 mg protein. About half of the protocols used an IDE [37, 40, 42], including two rush [38, 41] protocols that reached the maintenance dose in a few days. The protocols using an IDE built up to 30–140 mg EW protein on the first day, whereas the rush protocols built up to 1000–3000 mg EW protein over 1–5 days. Apart from rush protocols, most protocols built up every 1–2 weeks in clinic, although there were 4 protocols that included home build-up [36, 39, 40, 43]. Two studies using home build-up [36, 40] alternated lower (3–50%) build-up at home every 1–6 days with larger (30–100%) increases every 1–4 weeks in the hospital setting, whereas the other two [39, 43] protocols increased doses entirely at home. Two studies used very gradual home dosing (3–9% every 1–6 days) with more than 50 dose increases (up to 181) at home resulting in 80–100% of subjects reaching maintenance [40, 43]. One home-based protocol had subjects increase the dose more rapidly (25–100% every 3–7 days) with 56–69% of subjects reaching maintenance [39]. The study mentioned above that only enrolled patients with severe egg allergy increased by either 1 drop or 1 mL (10–50% dose increase) every 5–6 days at home, but none of the subjects was able to reach maintenance [36]. One study compared weekly observed build-up alone with small home-dose build ups between larger observed increases and found that more subjects on a combined clinic and home dosing reached maintenance (96% vs 75%, *p* = 0.01) in a shorter time (9 vs 18 weeks, *p* = 0.000) with lower rates of epinephrine use (3.8% vs 9.1%, *p* < 0.05) compared to weekly up-dosing alone [40].

Most protocols aimed to reach a maintenance dose over 3–10 months of approximately 1000–4000 mg of egg protein, or the equivalent of 1 egg, eaten every 1–3 days, although higher rates of success could be achieved over longer periods [42]. Two protocols used antihistamine premedication during build-up, including one rush protocol [38, 43]. Most subjects experienced AEs, including a 4–6% rate of anaphylaxis in most studies. One study with only low-dose home build up reported no cases of anaphylaxis [43]. Two studies reported higher rates of anaphylaxis [36, 40], but nothing about these studies stood out as factors affecting anaphylaxis, other than the one that only enrolled subjects with severe allergy, which reported 75% of subjects with Grade 3 or 4 reactions ^36^. Of the studies that reported chronic GI AEs, 3–9% of subjects dropped out due to these symptoms [37, 38, 41, 42].

### Milk OIT Protocols

Seven [44–50] RCTs are reviewed for CM OIT, and most were conducted in school-aged children although one enrolled infants 7–12 months of age [49]. Some studies excluded subjects who had a previous severe reaction to CM [45, 47], whereas others exclusively studied children with a history of anaphylaxis to CM [44, 50]. At baseline, most subjects reacted to < 1 mL (~ 33 mg protein) of CM, and all reacted to < 2 ounces (60 mL). Liquid CM was used for desensitization in most protocols, often diluted to 1:10, 1:25, or even 1:100, except for one study [45] which started with dry nonfat powdered CM and worked up to liquid CM. One study compared unheated CM with heated CM powder (prepared by heating CM at 125 °C for 30 s and spray-drying for 3 seconds) [50] and concluded that heated CM may have a better safety profile but may not induce as optimal desensitization [50].

The starting doses varied from 1 to 5 drops of diluted [44, 46–48] CM up to 1 mL undiluted [49, 50] CM (20 drops = 1 mL [48]). About half of the protocols started with an IDE [44, 45, 47, 49]. There was a wide range in terms of the IDE protocol: most completed the IDE in 5–10 doses over 1–2 days, up to a dose of 1–2 mL of undiluted CM. One RCT in children with severe milk anaphylaxis hospitalized subjects for 10 days to complete 36 doses starting with 5 drops of 1:10 CM dilution building up to 20 mL undiluted CM [44]. After the IDE (or instead of an IDE), 2 protocols reached maintenance in 8–9 doses [45, 50], 3 reported 16–18 steps [46, 47, 49], and 2 studies took > 100 doses [44, 48] to reach maintenance. A significant number of studies had subjects increase the dose at home as frequently as every few days to monthly [44, 48, 50]. One used a combination of home and observed dose increases [48]. The others increased the dose weekly in clinic then had subjects continue that dose at home daily [45–47, 49] except one which only dosed weekly in clinic, without any home dosing [46]. Only two [44, 50] of the 7 studies were premedicated with antihistamines. The majority of protocols reached maintenance in 2–6 months and two within 8–12 months [44, 50]. The majority of studies used 150–250 mL as the maintenance dose, and 2 used low [45, 50] doses of 3–15 mL for maintenance. The majority of studies had > 85% of subjects reach maintenance. Only one study, in subjects with severe anaphylaxis to CM, reported that most subjects failed to reach maintenance, even after a full year of build-up by 1 mL every other day at home [44]. Two studies [47, 48] reported high success (> 75% of subjects) consuming CM ad lib after OIT.

Most subjects experienced AEs associated with dosing. Five studies reported that > 75% of subjects experienced AEs from OIT [44, 47–49]. One study of home up-dosing only reported 20–32% of subjects with AEs [50]. The rate of anaphylaxis was 0–7% [44, 47, 48], except for in the studies with < 20 subjects [45, 46], in which the rate of anaphylaxis ranged from 20 to 33%. Only two studies reported subjects who developed chronic GI complaints, and this occurred in 1–3 subjects (3–11%) per study [48, 50]. One subject dropped out due to uncontrolled eczema [45].

### Wheat OIT Protocols

There has been one RCT [51], one high-dose rush [52], and a low-dose [53] observational protocol published for wheat OIT in children. The RCT excluded a history of severe anaphylaxis whereas, in the open-label studies, 94–100% of subjects had anaphylaxis to wheat. In the RCT conducted in the USA, vital wheat gluten was used since its high protein content allowed for smaller volumes than wheat flour. In the open-label Japanese studies, boiled udon wheat noodles were used as the investigational product.

All studies used an IDE of 8–10 doses over 1–5 days and then reached maintenance in 15–20 steps after the IDE (8 steps only for the low-dose protocol). The Japanese protocols used loratadine and montelukast as premedication and, after the 5-day IDE, all updosing occurred at home. The starting dose ranged from 0.07 mg wheat protein in the RCT to 2.8 mg in the low-dose and 50 mg in the high-dose protocols. Build-up occurred every 5–14 days and took a median of 20–40 weeks (up to 2 years in the high-dose observational study). The low-dose observational study built up to 53 mg, the high-dose protocol to 5200 mg, and the RCT to 1445 mg with the placebo arm crossing over to 2748 mg of wheat protein per day. More than 80% of subjects reached the maintenance dose, although only 57% of the placebo cross-over subjects attained high-dose maintenance in the first year, and some high-dose observational subjects required 2 years to reach maintenance.

In the RCT, up to 15.4% of doses during the first year were associated with AEs, which decreased to 3.1% of doses during the 2nd year of treatment. In the open-label studies, 26–30% of doses during the IDE were associated with AEs but only 4–6.8% of doses overall. The rate of anaphylaxis was < 0.05% of doses in all studies. Around 5–10% of subjects withdrew due to chronic abdominal pain or vomiting, except in the lowest-dose protocol in which no subjects withdrew.

After 2 years of 1445 mg OIT in the RCT, 13% achieved SU 8 to 10 weeks off therapy. In the open-label studies, 61–69% passed an OFC to their maintenance dose after 2 weeks off OIT.

### Tree Nut and Sesame OIT Protocols

There have not been any RCTs to individual tree nuts (TN), perhaps due to the complexity of cross-reactivity between different nuts. However, researchers in Israel have published a series of reports on OIT for walnut [54], cashew [55], and sesame [56] in which patients underwent an IDE over 1–2 days to identify their RD and then continued with 1–2 days confirming their MTD and repeating this process of dose escalation to a new MTD monthly for several cycles up to a goal of 4000 mg of the food protein. They showed that once reaching the 4000 mg dose, the daily dose could be decreased to 1200 mg, and patients could still tolerate a 4000 mg OFC after 6 months.

Recently, several observational reports of multi-food OIT for tree nuts have been published [6, 7]. One protocol conducted desensitization to each TN sequentially [6], whereas the other simultaneously dosed all relevant TN during a single multi-TN OIT build-up [7]. To simplify dosing for patients, one protocol [7] compounded the TN flour at local pharmacies into capsules or instructed patients to freeze TN milk into ice cubes which could be stored in the freezer for up to 3 months. At higher doses, patients could purchase a scale and weigh crushed whole TN. Although there were fewer patients undergoing multi-TN OIT than single-TN OIT in these studies, the rate of adverse reactions requiring epinephrine was not different for patients on multi-TN OIT versus single-TN OIT, suggesting that the safety of multi-TN OIT may be similar to single-TN OIT.

The two real-world [6, 7] studies started OIT with 1–10 mg of TN protein, whereas the Israeli group started with 0.1 mg. A retrospective report of hazelnut OIT started at 0.7 mg [57]. Most of the protocols used an IDE, with either 3 doses [6] given in a single day up to 3–10 mg of protein, or a 1–4 consecutive day IDE protocol [54–57] aimed at identifying the MTD up to 160–360 mg of protein. In these latter protocols, the MTD was then maintained for a month, and the protocol was repeated for several cycles until the 1635–4000 mg dose was achieved, then the dose was dropped to 416–1200 mg protein for maintenance. The other protocols [6, 7] built up in 25–100% increments every 2–4 weeks over approximately 6–7 months to a maintenance dose of around 300 mg protein. Given high cross-reactivity between cashew/pistachio and walnut/pecan, patients who underwent OIT for cashew or walnut could substitute doses using pistachio or pecan, respectively, either in clinic [7, 54, 55] or at home [6].

Higher rates of epinephrine use were reported during IDE and MDT protocols [6, 54–56] (0–25%), and with walnut, cashew, hazelnut, and sesame more than almond or macadamia nut, although there were only 4 reported subjects undergoing OIT for almond and 1 for macadamia nut. The rate of chronic GI AEs was similar as for other foods, and between single-TN and multi-TN studies, around 3–6%, when reported.

## Post-desensitization Strategies

Just as there is significant variability in the build-up phase of OIT, there is much uncertainty on the best strategy for patients after reaching the maintenance therapy phase of OIT. Similar to subcutaneous immunotherapy (SCIT) [58, 59], OIT has been shown to increase the percentage of regulatory T cells (Tregs) in an allergen-dependent manner [28] with downstream changes in allergen-specific IgE and IgG4, basophil activation and skin prick testing, and Th2 cytokines [28, 60], although tolerance with OIT remains to be proven. As a result, continued daily OIT is commonly recommended. Unfortunately, this can be difficult for families to sustain, and there is strong interest in more practical long-term options.

### Discontinuation of OIT

Several studies have shown the ability to achieve SU after discontinuing OIT for short periods of time up to 1 month [29, 42, 61, 62]. To understand whether long-term remission could be possible after the completion of OIT, the Immune Tolerance Network-sponsored IMPACT trial incorporated an extended 26-week OIT avoidance period after the completion of peanut OIT in 1–4-year-old peanut-allergic children [34]. After 134 weeks of peanut OIT with a 2000 mg maintenance dose, 71% of actively treated subjects achieved desensitization to 5000 mg compared to 2% of placebo-treated subjects. After discontinuing OIT for 26 weeks, 21% of active subjects again tolerated 5000 mg and were deemed to be in remission compared to 2% of placebo subjects. Post-hoc analysis identified lower age at OIT initiation and lower baseline peanut IgE as predictors of remission. In the clinic, these data suggest that most peanut-allergic patients will not be able to fully discontinue OIT outside of a subset of the youngest patients.

### Non-Daily Dosing Regimens

With consideration for the burden of OIT administration as well as the risk inherent with each exposure to food allergen, there would be a benefit to subjects with less frequent OIT dosing. In an extension of the pivotal PALISADE trial [25], the ARC004 study investigated every other day and twice weekly maintenance OIT dosing regimens in comparison to continued daily dosing [63]. The study found that desensitization rates declined on less than daily dosing with 72–92% of daily dosing subjects desensitized to 1000 mg peanut compared with 58–68% of non-daily dosing subjects. Daily dosing was not only superior but also longer durations of daily dosing appeared to result in increasing levels of desensitization. In addition, exposure-adjusted AE rates were higher in non-daily dosing regimens (25.95–42.49 AEs per subject-year) compared to daily dosing (12.94–17.54 AEs per subject-year). These data suggest that negative effects on efficacy and safety might be expected with non-daily dosing regimens at least when a 300 mg maintenance dose is utilized.

### Reduced Dose Maintenance Therapy

While full discontinuation of therapy has not appeared feasible for the majority of patients, the ability to transition to a lower dose of OIT would likely provide improved safety with the potential for less aversion and less treatment fatigue. In the POISED study, peanut-allergic subjects were treated with 4000 mg peanut OIT for 24 months, then either discontinued from OIT or maintained with a lower 300 mg peanut OIT dose for up to 12 months [35]. Similar to the IMPACT trial, remission was achieved by only 20% of subjects after 26 weeks and 13% of subjects 52 weeks after OIT discontinuation. Importantly, the reduction in maintenance dose to 300 mg also resulted in decreased efficacy. While 83% of subjects were fully desensitized, defined in the study as tolerating 4000 mg during OFC, after completing OIT, this percentage decreased to 54% 13 weeks after decreasing the maintenance dose and 43% after 26 weeks. These data suggest that lowering the OIT maintenance dose is likely to influence the degree of desensitization with a loss of desensitization expected for most.

### Dietary Food Equivalents

The use of peanut and other food flours for OIT has been convenient for dose measurement; however, these flours are not intended for direct ingestion. They require mixing with a vehicle food prior to eating and can result in taste and texture aversion. Transitioning to commercial food products with equivalent amounts of food protein to the OIT maintenance dose would simplify the dosing process and perhaps improve palatability as well. Specific to the FDA-approved Palforzia peanut OIT product, a food equivalent could also provide considerable cost savings. Groetch et al. recently published a simple-to-follow guide to selecting commercial food equivalents for varying dose levels of the most common food allergens [64]. As an example, ingestion of 300 mg of peanut protein could be achieved through 1.5 cocktail peanuts, ¼ tsp regular peanut butter, 3 Bamba snacks, or 4 Reese’s Pieces candies. Several key questions remain regarding a transition to dietary food products that warrant further study. First, it remains unclear if there is an ideal stage of treatment to transition from an OIT product to a food. Second, it is unknown whether equal amounts of protein in OIT and dietary food have equivalent effects on the immune system. Furthermore, a small study by Filep et al. suggested that protein content and ratios of the key peanut components may differ between different dietary options [65]. Overall, the transition to dietary food products should be jointly discussed with the patient considering factors such as reaction threshold, tolerance of OIT, adherence to treatment, cost, access, and patient preference.

## Conclusion

In summary, shared decision-making between the patient, family, and clinician must rely on balanced, evidence-based information to counteract the plethora of information families may encounter on social media. Decision-aids may supplement the one-on-one discussions physicians have with families. Many protocols have been published for milk, egg, and peanut OIT, and other foods to help guide physicians in providing evidence-based care for their OIT patients. A starting dose of around 0.1 mg protein, with or without an IDE, escalating every 2–4 weeks without premedication seem to be general trends across these protocols. Typical maintenance doses seem to cluster either around a low-dose 300–600 mg protein, or a high dose of 1200–4000 mg protein. Meta-analyses of the starting dose, steps to build-up, and maintenance dose would help further guide physicians to optimize the safety and efficacy of their chosen protocol. Upon achieving maintenance dosing, most patients will need to maintain exposure to the food allergen to remain desensitized. Less frequent or decreased OIT dosing can provide practical benefits but may affect desensitization. Switching to food products may have benefits for some patients. Further research is required.
